# SARS-CoV-2 Persistence and the Gut Microbiota: New Insights into Long COVID Pathogenesis

**DOI:** 10.3390/v18020247

**Published:** 2026-02-14

**Authors:** Sofia De Stefanis, Francesca Colavita, Fabrizio Maggi, Manuela Antonioli

**Affiliations:** 1 Ph.D. Program in Cellular and Molecular Biology, Department of Biology, University of Rome “Tor Vergata”, 00133 Rome, Italy; sofia.destefanis.01@alumni.uniroma2.eu; 2 Laboratory of Virology and Laboratories of Biosafety, National Institute for Infectious Diseases “Lazzaro Spallanzani”—IRCCS, 00149 Rome, Italy; fabrizio.maggi@inmi.it; 3 Department of Epidemiology, Preclinical Research and Advanced Diagnostics, National Institute for Infectious Diseases “Lazzaro Spallanzani”—IRCCS, 00149 Rome, Italy; 4 Department of Biology, University of Rome “Tor Vergata”, 00133 Rome, Italy

**Keywords:** SARS-CoV-2, gut microbiota, Long COVID

## Abstract

In December 2019, the world experienced the emergence of a new virus, SARS-CoV-2, which caused the 2020 pandemic. SARS-CoV-2 causes COVID-19, primarily affecting the respiratory system, as well as the gastrointestinal tract. Remarkably, one in eight COVID-19 patients develops Long COVID, which is linked to SARS-CoV-2 persistence in the gastrointestinal tract, resulting in chronic inflammation and microbiota dysregulation. Given that gut microbiota dysbiosis plays a pivotal role in antiviral defense and gastrointestinal conditions, here we examine emerging evidence on how persistent SARS-CoV-2 infection may contribute to the aetiology of enteric disorders. In particular, we emphasise the intricate connection between chronic inflammation caused by persistent SARS-CoV-2 infection (e.g., irritable bowel syndrome and inflammatory bowel disease) and the possible development of diseases such as Crohn’s disease and ulcerative colitis.

## 1. Introduction

The emergence of severe acute respiratory syndrome coronavirus 2 (SARS-CoV-2) at the end of 2019 led to a global pandemic in 2020, profoundly impacting global health, economies, and societies worldwide. Over 700 million people in the world have been infected, leading to more than 7 million deaths. SARS-CoV-2 viruses carry a positive-sense single-stranded RNA genome surrounded by an envelope [1] and have been described to enter the cells through the interaction between the viral protein Spike and the host receptor angiotensin-converting enzyme 2 (ACE2) [2]. Although the viral infection primarily occurs in the respiratory tract, ACE2 is expressed in several organs beyond the lungs; in particular, it is highly expressed in the epithelial cells of the gastrointestinal tract [3,4]. Indeed, betacoronaviruses such as SARS-CoV, MERS-CoV, and SARS-CoV-2 exhibit a characteristic intestinal tropism, with viral replication occurring in intestinal epithelial cells as a shared feature across multiple betacoronavirus lineages [5,6,7]. COVID-19, the disease caused by SARS-CoV-2 infection, is characterized by symptoms such as fever, cough, and nausea in the majority of cases. In the most severe forms, patients can show acute respiratory distress syndrome (ARDS) and multiple organ failure [8], which could eventually lead to death. Numerous studies indicate that gut dysbiosis is another consequence of SARS-CoV-2 infection [4,9,10,11,12]. ACE2 plays an essential role in the uptake of amino acids in the intestinal epithelium by controlling B0AT1, a co-transporter of Na^+^, along with neutral amino acids, such as tryptophan. Tryptophan is involved in the downregulation of lymphoid pro-inflammatory cytokines, the maintenance of intestinal tight junction integrity, the activation of antimicrobial peptide release, and the modulation of mucosal cell autophagy as part of the host defense mechanism, thus contributing to maintaining a proper coexistence between the host and beneficial commensals. The role of the ACE2–B0AT1 complex in intestinal amino acid transport and immune regulation has been firmly established in preclinical models, where ACE2 deficiency leads to impaired tryptophan absorption, altered antimicrobial peptide expression, and increased intestinal inflammation [13]. In humans, while ACE2 and B0AT1 are co-expressed in the small-intestinal epithelium [14] and COVID-19 is associated with systemic alterations in tryptophan metabolism [15], direct evidence of a persistent post-acute disruption of the intestinal ACE2–B0AT1–tryptophan axis remains limited and largely inferential. In this regard, it has been hypothesized that the impairment of ACE2 observed in SARS-CoV-2-infected cells, along with the subsequent downregulation of B0AT1, may affect tryptophan uptake and consequently alter the gut microbiota balance [4,16]. In line, Zuo et al. first demonstrated that patients with COVID-19 exhibited an altered microbiota. They described an increase in opportunistic microorganisms, such as *Coprobacillus*, *Clostridium ramosum*, and *Clostridium hathewayi*, which they correlated with a more severe disease outcome. Similarly, faecal microbiome analysis showed a decrease in bacterial species such as *Faecalibacterium prausnitzii*, *Eubacterium rectale*, and *Bifidobacteria*, which are beneficial for host immunity [12].

Although the emergency phase of the COVID-19 pandemic has been over for a few years now, its consequences continue to impact the global population. Indeed, based on an observational cohort study conducted in the Netherlands in 2022, one out of eight individuals affected by COVID-19 are affected by persistent symptoms, a multisystemic condition known as Long COVID [17]. Interestingly, it has been reported that vaccination before SARS-CoV-2 infection only partly reduces the risk of developing long-term symptomatic sequelae six months after COVID-19 [18]. Nevertheless, it must be acknowledged that incidence estimates vary greatly across studies due to differences in case definitions and the complexity of related symptoms. In the last few years, Long COVID has become a major, multifaceted challenge for global health. It affects several systems in the body, including the lungs, immune system, heart, and gastrointestinal tract [19]. Notably, some of these may be impacted despite low ACE2 expression [20]. Long COVID is characterized by a range of symptoms such as fatigue, brain fog, alteration of gut microbiota, and cardiovascular complications, among others. These symptoms typically begin around three months after the initial infection and persist for at least two months [21]. The development of this secondary condition has been associated with persistent SARS-CoV-2 viral infection in the host’s gastrointestinal tract. Indeed, several studies reported the presence of viral RNA, protein fragments, and SARS-CoV-2 antigens in the gut of patients long after their recovery from the acute phase of COVID-19, highlighting the heterogeneity of findings among studies and suggesting that the gut microbiota may influence viral decrease and the disease’s outcome [22].

## 2. Gut Microbiota and Its Interaction with Gastroenteric Viruses

The human microbiota comprises a wide variety of organisms, including viruses, protozoa, bacteria, and fungi, that inhabit specific sites in the body, such as the skin, oral cavity, lungs, and gut. The host and commensal microorganisms have evolved a relationship of mutual benefit [23,24]; in fact, dysregulation of this equilibrium has been linked to the onset of various diseases [23]. The microbiota is not static; it is in a state of dynamic equilibrium and modifies in response to external stimuli [25]. The gastrointestinal tract harbours the largest population of human microbiota, with approximately 10^13^ distinct commensals, which mainly reside in the distal intestinal tract due to factors such as pH, redox potential, oxygen levels, mucin secretion, and nutrient availability [24,26]. Six main phyla are present, including *Actinobacteria*, *Bacteroidetes*, *Firmicutes*, *Fusobacteria*, *Proteobacteria*, and *Verrucomicrobia* [23], and, depending on the characteristics of each individual and their environment, the proportions of specific bacterial types may vary [24]. In this scenario, maintaining a balanced gut microbiota is essential [23], as disruptions in this delicate equilibrium can lead to immune dysregulation and chronic inflammation. These alterations can cause gastrointestinal alterations such as IBD and IBS [27,28], which could subsequently progress to Crohn’s disease or ulcerative colitis [27]. Furthermore, the microbiota has a crucial role in viral infections: it can serve as a physical barrier to viral particles, and commensal bacteria can prevent infection by enhancing host defenses. Nevertheless, some enteric viruses have evolved alongside the microbiota and utilize the bacteria to bolster the infection [24,28] (Table 1). For example, it has been described that some enteric viruses, such as *Rotavirus* and *Poliovirus*, are assisted by gut commensal bacteria in infecting host cells [29,30]. In detail, while Polioviruses specifically bind to the lipopolysaccharides (LPS) of Gram-negative bacteria, Rotaviruses, like other enteric viruses, interact with both Gram-positive and Gram-negative bacteria through mechanisms that are still unclear [29]. The association between the virus and the bacteria stabilizes the viral particles [31], favouring their binding to the host cell and, consequently, promoting co-infection [29].

Differently, the gut microbiota also exerts an inhibitory effect on infections. On the one hand, nutrient competition creates specific ecological niches that favour beneficial bacteria. On the other hand, the gut microbiota itself produces various molecules, such as antimicrobial peptides, short-chain fatty acids (SCFAs), and other bioactive compounds, which play a critical role in host immune regulation. SCFA (mainly acetate, butyrate, and propionate) and bile acids together promote anti-inflammatory pathways by stimulating Treg cell differentiation and activating IL-10, thereby downregulating TNF-α and pro-inflammatory responses. Ultimately, reducing the inflammation produced by the infection, preventing autoimmunity, and reducing mucosal damage. Additionally, butyrate induces mucin production and strengthens intestinal epithelial tight junctions, enhancing barrier integrity and mucosal immunity [27]. Thus, the complex interplay between the gut microbiota and enteric viruses plays a crucial role in regulating both viral infection and host immune responses.

## 3. The Effects of Microbiota in Long COVID

**Figure 1:**
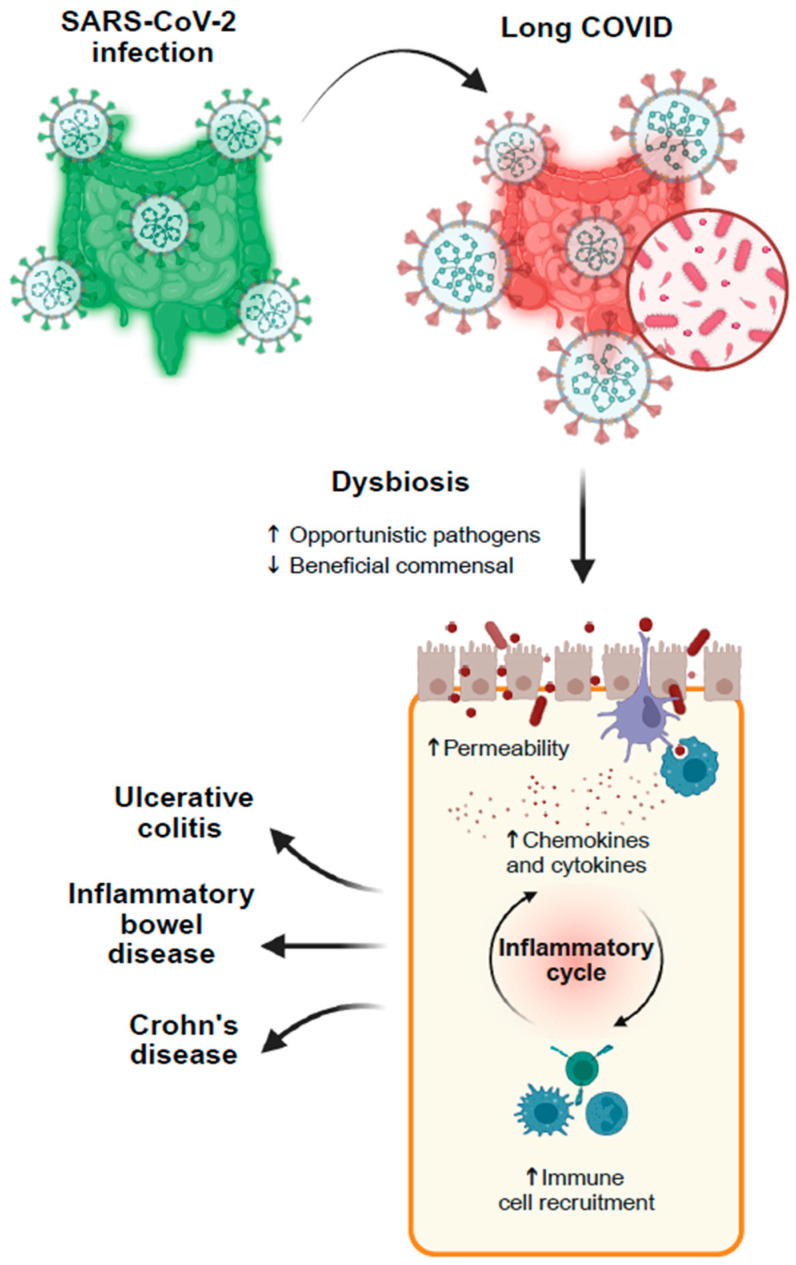
Gut microbiota in Long COVID outcomes: a hypothetical model. Infection with SARS-CoV-2 is associated with alterations in the gut microbiota. The reduction of beneficial commensals and the increase in opportunistic microorganisms lead to dysbiosis; this alteration of the microbiota increases intestinal permeability, facilitating the translocation through the gut barrier of viral and microbial products. Furthermore, it triggers the release of cytokines and chemokines, thus activating pro-inflammatory pathways and recruiting immune cells. This perspective hypothesizes that the prolonged dysbiosis and chronic inflammation associated with Long COVID may increase the risk of developing more serious conditions like inflammatory bowel disease, ulcerative colitis and Crohn’s disease. https://BioRender.com/23k6xd7 (accessed on 8 February 2026).

Recently, it has been shown that SARS-CoV-2 infection can alter the gut microbiota, especially during prolonged infection, which may develop into Long COVID [31,46,47]. Features such as immune dysregulation and chronic inflammation, which are most common in Long COVID patients, associate the infection with dysbiosis and highlight the role of the microbiota in the progression of the disease [22] (Figure 1).

It has been clearly shown that SARS-CoV-2 in the gastrointestinal tract disrupts the equilibrium of commensal bacteria in the intestine, contributing to the development of symptoms such as diarrhoea, nausea, vomiting, and abdominal pain that are common in COVID-19 cases [9,11]. Invasion of SARS-CoV-2 activates pathways of both innate and adaptive immune responses, including NF-κB and JAK/STAT signalling. The resulting inflammatory state caused by the infection negatively affects the gut microbiota [9]. As mentioned above, various studies have correlated symptom severity with a higher level of dysbiosis. The increase in specific bacterial strains, such as *Bacteroides*, *Clostridium*, *Rothia* and *Streptococcus* has been observed in hospitalized COVID-19 patients compared to controls. Members of the genus *Coprobacillus*, such as *Clostridium* bacteria, have been observed to upregulate ACE2 expression, correlating with a stronger SARS-CoV-2 infection. On the contrary, butyrate-producing bacteria such as *Faecalibacterium prausnitzii* are present in lower amounts, thereby increasing pro-inflammatory pathways [9,11,12].

Of interest, the intestinal microbial population interacts with the central nervous system (CNS) via the gut–brain axis [48,49]. The gut-associated lymphoid tissue (GALT) plays a fundamental role in this axis and, by interacting with the gut microbiota, further influences the maturation and activity of various immune cells, thereby maintaining immune tolerance in individuals [22,50]. Exploiting this link, the SARS-CoV-2 virus could potentially impact the nervous system, causing symptoms like brain fog and cognitive deficits that are typical during Long COVID. The virus-induced dysbiosis can disrupt the integrity of the epithelial barrier, and during inflammation, immune cells might easily enter the circulation and potentially gain access to the CNS. In line with this hypothesis, higher levels of cytokines and chemokines have been found in the cerebrospinal fluid of a wild-type mouse model administered with adeno-associated virus expressing hACE2 after SARS-CoV-2 infection, indicating that the dysbiosis-produced inflammation might be involved in the cognitive symptoms observed in COVID-19 and Long COVID [51]. In a longer-term perspective, gut immune dysregulation associated with COVID-19 may be linked to an increased susceptibility to gastrointestinal disorders, such as IBS and inflammatory bowel diseases, including Crohn’s disease and ulcerative colitis [52,53]. However, these associations should be interpreted cautiously, as current evidence does not support a direct progression from Long COVID–associated gut inflammation to chronic inflammatory diseases or colorectal cancer, which typically arise after long-standing inflammation and long latency periods. To date, it has been established that COVID-19 severity is associated with an increased risk of serious long-term health consequences, and a recent meta-analysis involving 3998 patients reported that individuals infected with SARS-CoV-2 had a six-fold higher risk of developing IBS and an eight-fold higher risk of developing dyspepsia compared with non-infected controls [54]. Similarly, several case reports have shown an increased risk of *de novo* Crohn’s disease [55,56]. In the case described by Senthamizhselvan et al., a 33-year-old woman affected by mild COVID-19 shortly after the infection developed symptoms such as large volume, watery, and non-bloody diarrhoea associated with periumbilical pain. Over the next few weeks, she also presented with ulcers in the colon, leading to the diagnosis of *de novo* Crohn’s disease [55]. However, further longitudinal studies are required to clarify whether these conditions represent true long-term sequelae or shared risk associations. Another possible outcome of the infection may be the manifestation of ulcerative colitis, as illustrated in multiple studies, although the available evidence remains limited and correlative. Symptoms like abdominal pain and bloody diarrhoea have been described starting between one week and four months post-infection, more likely in patients who previously experienced gastrointestinal symptoms during COVID-19 [57,58], suggesting that SARS-CoV-2 virus may contribute to the development of gastrointestinal conditions in susceptible individuals. However, the underlying mechanisms remain insufficiently understood, partly due to confounding factors affecting patients, such as antibiotic exposure, diet, and hospitalization, and therefore require further investigation.

It is essential to note that dysbiosis of the microbiota has also been described as a risk factor for the possible development of colorectal cancer (CRC), which typically arises after prolonged periods of long latency. Patients presenting long-standing inflammatory diseases such as IBD and chronic colitis are at a higher risk of developing a specific type of CRC, colitis-associated cancer (CAC) [59]. CRC is the third most common cancer and the second leading cause of cancer-related death in both females and males, with CRC cases increasing globally in recent years and expected to continue rising [60]. In this context, we speculate that chronic gastrointestinal inflammation associated with Long COVID might represent, in the long term, one of several complex factors involved in the processes underlying CRC development.

Currently, there is not enough evidence to support this hypothesis linking Long COVID associated gut inflammation and an increased risk of developing colorectal cancer (CRC). Therefore, further studies are necessary to better understand this potential association, especially considering that CRC typically develops after long-term chronic inflammation. Moreover, given the multifactorial nature of Long COVID and its complex symptomatology, distinguishing it from other syndromes is particularly challenging. Therefore, it will be of fundamental importance to identify specific biomarkers that facilitate diagnosis.

## 4. Conclusions

Since 2019, the appearance of a new virus has radically impacted the globe. In recent years, it has been observed that SARS-CoV-2 infection can persist in the gastrointestinal tract even after COVID-19 symptoms resolve, leading to the condition called Long COVID. The virus reduces beneficial commensal bacteria and increases opportunistic microorganisms, leading to chronic inflammation and dysbiosis. The role of the gut microbiota in regulating viral infections and the immune system is well recognized. Moreover, gut dysbiosis has been implicated in inflammatory bowel disease, and long-standing IBD is associated with an increased risk of colitis-associated colorectal cancer. In this context, preliminary evidence, mainly from case report studies, suggests a potential association between SARS-CoV-2 infection and the new onset of the mentioned gastrointestinal diseases. To date, these findings are heterogeneous and observational, and any implications for colorectal pathology or cancer should be interpreted cautiously given the long latency and chronic inflammatory processes typically required. Nevertheless, in light of the evidence discussed above, dedicated studies may be warranted to evaluate a potential association between Long COVID and the incidence of inflammatory and neoplastic colorectal diseases. In addition, the molecular mechanisms underlying the interaction between SARS-CoV-2 and alterations in the gut microbiota remain poorly understood. Therefore, further research is essential to clarify these mechanisms and, more importantly, to facilitate earlier and more accurate diagnostic approaches, thus reducing the risk of dysbiosis and chronic intestinal inflammation.

## Figures and Tables

**Table 1:** Microbiome- and Gut Environment Modulation during enteric viral infections.

Biological Process	Effect	Virus	Reference
**Direct interaction**			
**Infection of intestinal epithelial cells**	Epithelial barrier disruption and increase in gut permeability, altering local immune responses and nutrient absorption	SARS-CoV-2 Rotavirus Norovirus Poliovirus	[32,33,34,35]
**Binding to bacterial products (LPS and HBGA-like structures)**	Stabilization of the viral capsid, enhancement of the receptor binding and infection, skewing host immune response; Support of the transmission and thermostability	Rotavirus Norovirus Poliovirus	[34,36,37,38,39]
**Microbiome-related mechanism**			
**Microbiome** **remodelling**	Marked dysbiosis, mostly with: - depletion of *Lactobacillus*, *Faecalibacteriumi*, *Eubacterium* and *Bifidobacterium*, - enrichment of Gram-negative bacteria with mucin-degrading capacity (e.g., *Bacteroides, Proteobacteria, and Akkermansia*)	SARS-CoV-2 Rotavirus Norovirus Poliovirus	[32,34,36,37,38,40]
**Host immune mechanism**			
**Modulation of innate immunity**	Reduced intestinal IL-22 expression, with the impairment of the viral control and contribution to the damage of the gut barrier	Rotavirus Norovirus Poliovirus	[34,38,39,41,42]
**Inflammatory state**	Epithelial barrier disruption and increase in the gut permeability	HIV Influenza virus SARS-CoV-2	[40,42,43,44,45]

## Data Availability

The original contributions presented in this study are included in the article. Further inquiries can be directed to the corresponding authors.
